# Learning from 2523 trauma deaths in India- opportunities to prevent in-hospital deaths

**DOI:** 10.1186/s12913-017-2085-7

**Published:** 2017-02-16

**Authors:** Nobhojit Roy, Deepa Kizhakke Veetil, Monty Uttam Khajanchi, Vineet Kumar, Harris Solomon, Jyoti Kamble, Debojit Basak, Göran Tomson, Johan von Schreeb

**Affiliations:** 1 Health Systems and Policy, Department of Public Health Sciences, Karolinska Institutet, Stockholm, Sweden; 2 Department of Surgery, Bhabha Atomic Research Centre Hospital, Mumbai, India; 3 School of Habitat, Tata Institute of Social Sciences, Mumbai, India; 4 Department of Surgery, King Edward Memorial Hospital, Mumbai, India; 5 Department of Surgery, Lokmanya Tilak Municipal Medical College and General Hospital, Mumbai, India; 6 Department of Cultural Anthropology and Global Health, Global Health Institute, Duke University, 205 Friedl Building, Box 90091, Durham, 27708 NC USA; 7 Department of Learning, Informatics, Management and Ethics (LIME) and Public Health Sciences, Karolinska Institutet, Stockholm, Sweden

## Abstract

**Background:**

A systematic analysis of trauma deaths is a step towards trauma quality improvement in Indian hospitals. This study estimates the magnitude of preventable trauma deaths in five Indian hospitals, and uses a peer-review process to identify opportunities for improvement (OFI) in trauma care delivery.

**Methods:**

All trauma deaths that occurred within 30 days of hospitalization in five urban university hospitals in India were retrospectively abstracted for demography, mechanism of injury, transfer status, injury description by clinical, investigation and operative findings. Using mixed methods, they were quantitatively stratified by the standardized Injury Severity Score (ISS) into mild (1–8), moderate (9–15), severe (16–25), profound (26–75) ISS categories, and by time to death within 24 h, 7, or 30 days. Using peer-review and Delphi methods, we defined optimal trauma care within the Indian context and evaluated each death for preventability, using the following categories: Preventable (P), Potentially preventable (PP), Non-preventable (NP) and Non-preventable but care could have been improved (NPI).

**Results:**

During the 18 month study period, there were 11,671 trauma admissions and 2523 deaths within 30 days (21.6%). The overall proportion of preventable deaths was 58%, among 2057 eligible deaths. In patients with a mild ISS score, 71% of deaths were preventable. In the moderate category, 56% were preventable, and 60% in the severe group and 44% in the profound group were preventable. Traumatic brain injury and burns accounted for the majority of non-preventable deaths. The important areas for improvement in the preventable deaths subset, inadequacies in airway management (14.3%) and resuscitation with hemorrhage control (16.3%). System-related issues included lack of protocols, lack of adherence to protocols, pre-hospital delays and delays in imaging.

**Conclusion:**

Fifty-eight percent of all trauma deaths were classified as preventable. Two-thirds of the deaths with injury severity scores of less than 16 were preventable. This large subgroup of Indian urban trauma patients could possibly be saved by urgent attention and corrective action. Low-cost interventions such as airway management, fluid resuscitation, hemorrhage control and surgical decision-making protocols, were identified as OFI. Establishment of clinical protocols and timely processes of trauma care delivery are the next steps towards improving care.

## Background

Ninety percent of global trauma mortality occurs in low-and-middle-income countries (LMICs) [1]. In high income countries (HICs), trauma mortality has steadily declined, but a similar trend is not seen in LMICs [2]. In India, the trend of in-hospital trauma mortality has remained unchanged in the past decade, despite advances in imaging and medical equipment [3]. In addition, India’s 30-day trauma mortality rate is twice that of comparable patients admitted to trauma centres in HIC settings [4]. The reasons for the high rates and unchanging trends remain unknown and unexplored [4]. High clinical load [5], low-resources, and high out-of-pocket expenditures [6] are commonly named as barriers to improving trauma care in India [7]. However, several studies have demonstrated that low-cost interventions can improve trauma care outcomes [1, 8].

If hospitals provide mortality data in relation to trauma care outcomes, risk-adjusted death rates can be used to compare outcomes among different countries [2]. A higher trauma mortality rate calls for attention to the factors that contribute to the deaths [4, 9]. A systematic analysis of all trauma deaths, in order to identify preventable trauma deaths, is recognized as the first step towards trauma system improvement [10–12]. Peer-review and trauma audit filters are established tools for evaluating and monitoring trauma care systems [13]. Suboptimal trauma care is preventable, and this has led to the development of trauma systems in HICs.

Both the preventable deaths rate and risk-adjusted mortality rate are used to measure trauma system performance between institutions and countries. The proportion of preventable deaths of all in-hospital trauma deaths ranges from less than 20% in HIC countries [14], to more than 60% in LMICs like Brazil [12] and Ghana [15]. However, the proportion of preventable deaths within overall trauma mortality is not known in India [4]. The objective of this study was to estimate the proportion of preventable deaths. The secondary objective was to identify OFI in trauma care delivery for preventing deaths in the context of urban university hospital in India.

## Methods

Study population: Five university hospitals participated in the study, and were located in megacities with populations of more than 10 million, to represent urban India. The hospitals were the Apex Trauma Centre of the All-India Institute of Medical Sciences (AIIMS), New Delhi; Lokmanya Tilak Municipal General Hospital (LTMGH), Mumbai; King Edward Memorial (KEM) Hospital, Mumbai; Rajiv Gandhi General Hospital, Chennai; and the Seth Sukhlal Karnani Memorial Hospital (SSKM), Kolkata. All five hospitals are classified as ‘free-to-public’, indicating nominal fees to users facilitating access to care to the lower socio-economic strata of the population, and operate high-volume trauma units that receive city-wide trauma patient referrals. The differing processes and infrastructures in each of the participating hospitals without identifying them, are outlined in detail in a previous paper [16].

The data for this prospective, multicentre, observational cohort study was systematically collected by trained data collectors, under the guidance of the collaborative research consortium “Towards improving trauma care outcomes” (TITCO-India). The method has been previously described in detail [4]. The study period was from August 1, 2013 to February 28, 2015.

All deaths among hospitalized trauma patients within 30 days of admission were retrospectively abstracted (by author NR) from the collected data, and included information on demographics, mechanism of injury, transfer status, injury description by clinical investigation and operative findings, injury severity score, and time to death [4]. No prehospital information was available, as there was no formal prehospital care or transport in the settings. Since 30-day mortality was the primary outcome, patients who died after 30 days or whose case records did not have sufficient information to allow death review were excluded. The included mechanisms of injury were mechanical or thermal injury; poisonings and drownings were excluded.

**Fig. 1:**
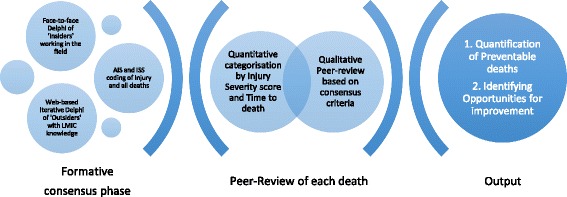
Sequential mixed-method for generation of consensus criteria for contributors to all trauma deaths
- *Formative phase of consensus building*: There is a lack of data about the causes of trauma deaths in India [17, 18]. Therefore, the formative phase was started by selecting a Delphi panel of Indian trauma care health providers with at least 2 years’ of trauma care experience in the local context. This ‘insider’ panel included four trauma surgeons, a trauma researcher, and a medical anthropologist (male to female ratio [M:F] = 2:1). The national panel of six experts met face-to-face and used an iterative Delphi process, to reach consensus about the contributors to deaths in trauma patients, and what constitutes optimal care for preventable deaths in the Indian context. The contributors and optimal care factors were based on experience and prior biological knowledge about resuscitation, trauma care protocols, and airway, surgery or long-stay complications. For the international perspective, an ‘outsider’ panel was formed. Nine international trauma experts with experience of working or observing in LMICs were invited to join, of which six (M:F = 2:1) consented. These six international experts completed an anonymous web-based Delphi session, to independently prioritize the contributors to death, scored between one and ten, from least to most relevant. Clarifications were then sought for each contributor to death, and the panel reached consensus through a blinded, iterative process.
- *Peer-review phase*: Using the consensus findings of the Delphi panels, three panelists of the Indian panel group were trained in the peer-review process of trauma deaths [19] for five hours by the first author (NR), in order to be able to describe and define actions or events which could have contributed to or prevented deaths. This was followed by a practice session with mock cases taken from the WHO guidelines for trauma quality improvement [13].

- *Design*: A sequential mixed-method design was used to address quantitative and qualitative questions as shown in Fig. 1. The final output is an estimation of the proportion of preventable deaths among all trauma deaths, using the WHO classification of preventability [13], and identified OFI of trauma care in India.

In keeping with international standards [20], quantitative benchmarking of in-hospital mortality was achieved by stratifying the death dataset by the Injury Severity Score (ISS) (by authors, DKV, JK, DB). Deaths were categorized into mild (1–8), moderate (9–15), severe (16–25), or profound (26–75) ISS categories, and by time-to-death within 24 h, 7, or 30 days (by author NR). Thereafter, each death was evaluated for preventability, using the following categories: Preventable (P), Potentially preventable (PP), Non-preventable (NP) and Non-preventable but care could have been improved (NPI) [13] by authors NR, DKV, VK, MUK). Deaths were further evaluated for a probable cause of death, in order to identify a broad area of improvement. Of the total deaths, 466 (18.4%) deaths were excluded because they were misclassified, had inadequate documentation or the cause could not be determined.

## Results

**Fig. 2:**
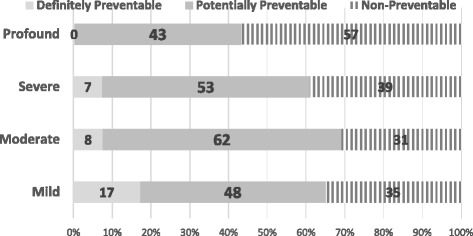
Proportion of preventable deaths among all trauma-deaths (*n* = 2057)

During the 18-month study period, there were 11,671 trauma admissions and 2523 deaths within 30 days (21.6%) in the five urban university hospitals. A total of 2057 deaths were eligible for analysis (81.6%). Of these, 233 (11%) were classified in the mild ISS group, 922 (45%) in the moderate group, 571 (28%) in the severe group, and 331 (16%) in the profound ISS category group. The overall proportion of preventable deaths was 58%. Table 1 summarizes the time-to-death of all the deaths, categorized by injury severity. After peer-review, the proportion of preventable deaths in patients with mild ISS was 71, 56% in the moderate category, 60% in the severe category and 44% in the profound ISS category. As shown in Fig. 2, more than two-thirds of deaths among not seriously injured subgroups, with an ISS score of less than 16, were considered preventable.

**Table 1:** Timing of in-hospital deaths classified by injury severity

Severity	Early (<24 h)	Delayed (1–7 days)	Late (8–30 days)	Total
Trauma deaths	601 (29%)	960 (47%)	496 (24%)	*n* = 2057
Mild (ISS < 9)	116 (50%)	67 (29%)	50 (21%)	233 (11%)
Moderate (9–15)	265 (29%)	455 (49%)	202 (22%)	922 (45%)
Severe (16–25)	124 (22%)	277 (48%)	170 (30%)	571 (28%)
Profound (>25)	96 (29%)	161 (49%)	74 (22%)	331 (16%)

The consensus of contributing factors for trauma deaths, produced by the national and international panels, are presented in Table 2. The problem areas ascertained were resuscitation, lack of trauma care protocols, and airway, surgery and long-stay complications. The consensus on early causes of death were haemorrhage, inadequate fluid resuscitation, and inadequate airways. The late contributors to death were systemic factors, ventilator management, disseminated intravascular coagulation (DIC) and sepsis.

**Table 2:** Why do trauma patients die? Exploring contributors to death by Delphi consensus by national and international panel of experts

	Least to most relevant Score 1–10	
	International experts	Indian researchers
*1. Where there is no system of trauma care, with the existing available resources & information, the following are the most probable reasons for in-hospital trauma deaths in India:*		
Delayed control of haemorrhage-Intra-abdominal and pelvic Haemorrhage	10	8
Delayed control of haemorrhage-Intra-thoracic Haemorrhage	10	8
Delayed control of haemorrhage-Extremity Haemorrhage	5	6
Delayed resuscitation	10	10
Inadequate resuscitation	10	10
Lack of blood	7	10
Inadequate monitoring of vitals	8	10
*2. With poor GCS, the probable reasons why trauma patients with poor GCS die early within days are:*		
Overwhelming impact of Traumatic Brain injuries	8	8
Uninvestigated Head injury	8	2
Untreated Head Injury-no surgery done	8	6
Inadequate airway management	6	10
Inadequate ventilatory management	10	10
Decision not to operate-left to die	6	5
*3. Systemic issues which contribute to death in trauma patients in urban Indian hospitals:*		
Prehospital delay contributing to in-hospital mortality	8	8
Lack of basic investigations	6	4
Lack of advanced imaging facilities	2	4
Lack of ventilator	6	10
Unduly long surgery done	2	4
Inappropriate surgery done	4	6
Unstable patient operated on	4	3
Unstable patient sent for CT or USG	4	6
Lack of protocols	8	10
Lack of adherence to protocols	10	8
*4. The long-term reasons why trauma patients die in the weeks following admission are:*		
Quite unknown	10	8
Sepsis	8	8
Ventilator related complications	3	10
Pneumonia	8	10
DIC	2	4

The peer review of deaths found that severe traumatic brain injury and burns over more than 80% total body surface area accounted for the majority of non-preventable deaths. In the preventable deaths subset (Table 3), inadequacies in airway management (14.3%) and resuscitation after hemorrhage (16%) were the most common reasons for death. System-related issues included lack of protocols, lack of adherence to protocols, and pre-hospital delays in arrival for care. Inappropriate surgical decision making, unsuitable surgeries, and ill-timed long surgeries were contributing factors in 3.5% of deaths.

**Table 3:** Opportunities for improvement in the preventable group of deaths as identified by peer-review

Opportunity for improvement	*n* = 2057	Percentage
Resuscitation related:		
Delayed control of pelvic abdominal hemorrhage	51	2.5
Delayed control of intrathoracic hemorrhage	25	1.2
Delayed resuscitation	108	5.2
Inadequate resuscitation	152	7.4
Lack of blood	11	0.5
Protocol lack or lack of adherence:		
Intensive monitoring required	109	5.3
Unavailability of ventilator	39	0.1
Unstable patient sent to CT	9	0.8
Inappropriate ventilatory management	1	1.9
Delay in cervical spine	9	0.4
Left to die	49	2.4
Prehospital delay	212	10.3
Unknown cause	4	0.2
Lack of investigations	11	0.5
Lack of protocols	24	1.2
Lack of adherence to protocols	4	0.2
Head Injury and Airway related:		
Airway	293	14.3
Traumatic Brain injury	307	14.9
No CT uninvestigated	56	2.7
Untreated head injury	64	3.1
Untreated head injury-no surgery done	109	5.3
Surgery related:		
Inappropriate surgery done	5	0.2
Unduly long surgery done	61	3.0
Unstable patient operated on	5	0.2
Negative explorations	1	0.1
Injury prevention:		
Burns prevention	117	5.7
Long stay complications:		
DIC	1	0.1
Sepsis	39	1.9
Pneumonia	16	0.8
Old age related complications	19	0.9
Ventilator related complications	104	5.1
Miscellaneous issues:		
Could not be determined	26	1.2
Misclassified	5	0.2

## Discussion

In this study, more than half of the in-hospital trauma deaths were preventable. Estimating the magnitude of this previously unknown rate fills a trauma care knowledge gap for India. Our preventable trauma death rate is similar to two other LMICs, with rates of 40 to 60% [15, 17], but much higher than HICs, which range from 4 to 20% [14, 21]. Quantifying the preventable deaths prompted the identification of OFI to bridge this gap, based on problem identification in the urban trauma centres.

It is noteworthy that the peer-review of the deaths determined that there was a proportion (17%) of mildly injured, but dead, patients. Perhaps, the underlying fatal injuries in this mild subgroup (ISS < 9) were underestimated as patients were uninvestigated or their CT imaging was not documented. These issues were not always under the direct control of the clinical team, but would be important contributors to failures of treatment and care. A thorough investigation to discover covert and potentially fatal injuries, as part of a future targeted intervention, could save many lives in this subgroup with seemingly mild injuries [22].

Inadequate fluid resuscitation and hemorrhage control were the leading causes of death [23] among definitely preventable deaths. Inadequate fluid resuscitation was a common problem found in other similar LMIC studies [15]. The Advanced Trauma Life Support (ATLS) course or similar training initiatives are likely to improve the understanding of surgical physiology of the injured and the body’s response to trauma. These initiatives cover low-cost, protocol-based interventions that include the placement of multiple large-bore intravenous access, use of hypertonic solution in the resuscitation of hypotensive patients [22], and early use of analgesics [24].

Developing context-specific standard treatment protocols based on best practice and damage control resuscitation models [22, 25] are recommendations to reduce deaths. While the lack of adherence to protocols leads to failures even in HICs [26], the frequency is higher in LMICs [15], where the protocols are not defined and implemented.

In patients who underwent surgical interventions, preventable deaths were associated with surgical judgement. There were delayed, prolonged and inappropriate surgeries [27]. However, there was a subgroup of patients, in whom the decision was ‘not to operate’, as it was decided by consensus that medical intervention would be futile in the local setting. These were identified as ‘left to die’. Though a harsh label, ‘left to die’ [28] usually signified an appropriate decision made by the treating trauma team, based on the local resources.

Though lack of resources dominates most conversations about the challenges facing LMIC trauma care, several studies suggest that low-cost interventions, protocols and systems for supplies may be more beneficial than the mere addition of high-cost and mismatched resources [15, 29]. Overall improvements in the trauma system in India will begin with the adoption of appropriate actions as process guidelines, as demonstrated effectively by a modest Thai hospital in Khon Kaen [30]. The presence of an attending surgeon [31], a trauma team leader during resuscitation [32], the initiation of academic trauma management programs [33], and grand rounds [8] via teleconference [34] have all been shown to beneficially impact the rate of preventable deaths.

An improvement in Indian trauma care can begin by shifting the focus away from the individual providers and their errors to a system-wide perspective. Non-clinical processes of healthcare delivery were identified in this study as an important contributor to trauma mortality. In a previous study [35], the authors have described delays and identified the process of care indicators for correction. Many systems-related issues, such as suboptimal multidisciplinary collaboration and lack of a trauma leader, require moderated, multidisciplinary mortality and morbidity meetings. WHO-recommended preventable death panel meetings can also improve system-related issues. These meetings must be viewed as opportunities, and should adopt the Avedis Donabedian approach of destigmatizing the individual as a target to “blame” for unfavorable outcomes [36]. This requires a wider mix of participants on these committees, like patient representatives or administrative staff, who will address aspects of care beyond standard surgical and clinical aspects.

Since prior scientific knowledge about in-hospital trauma deaths in India was unavailable, the study was designed to be a mixed methods exploratory study, though cumbersome and with limitations. Triangulation was achieved when more than two experts agreed on a particular cause of death or opportunity for improvement in the Indian context. Initially, the multidisciplinary preventable death panels were piloted as advocated by the WHO [13] at three participating sites, with varying success. After that learning experience, the more empirical Delphi method of expert multidisciplinary consensus was chosen.

There are acknowledged limitations of the study. Errors and adverse events could not be captured, and this will require more sophisticated systems [37] of recording and diagnosis. With the given information, neither the exact cause of death nor a root cause analysis for trauma quality improvement was feasible in this study. Approximately a fifth of the deaths (18%) could not be evaluated, due to inadequate information, documentation or investigations. Second, since there is no formal prehospital system in India, the OFIs and errors during that phase of care were unavailable. This phase contributed to half of the OFIs identified in HIC centres [21]. Deaths that occurred before reaching the hospital and also after discharge are missing in the dataset. Therefore, this study of in-hospital deaths represents only a part of the whole trauma picture; it also excludes trauma in rural India [19]. Third, the determination of preventable deaths (definitely, potentially preventable) is subjective in all similar studies, especially across institutions and countries [15]. Therefore, the inter-rater variability or reliability among the reviewers was not calculated in this Indian registry, as inconsistency is acknowledged even in the comprehensive HIC trauma registries.

Retrospective judgments on clinical decision-making, based on case record findings, must be examined with extreme caution, and this study has been careful to use only objective parameters, like prolonged operative time, or pre-operative physiological status. Perceptively, the Delphi consensus group noted that there were unobserved factors like DIC that may have contributed to death, but the peer-review panel could not attribute them as causes since they were not documented in the case records nor were autopsy findings available [38]. Other studies have noted issues such as missed injuries, nosocomial pneumonia, surgical site infection, pulmonary embolism, deep vein thrombosis, alcohol use, acute respiratory distress syndrome, gastrointestinal ulcers, pericardial tamponade, hyperkalemia, unintended extubation, intravascular catheter related complications, overdose, air embolism and mismatched transfusion [39, 40]. These factors would require more systematic research, before their contribution to trauma deaths is determined in the Indian context.

If the results remain valid in other Indian hospitals, it is worth noting that better identification and management of trauma patients could save a quarter of a million lives each year, based on a 50% reduction of the estimated half a million annual hospital trauma deaths in India.

Additionally, to reach out beyond in-hospital trauma care, there are visionary interventional strategies provided by the American College of Surgeons Committee on Trauma (ACS/COT) that include leadership, system development, legislation, finances, injury prevention, human resources [41], pre-hospital care, definitive care facilities, information systems, evaluation, disaster preparedness research [42] and maintenance of a trauma registry [43]. With implementation of these progressive steps, India can reduce preventable deaths through a standardized reporting of preventable errors and analysis of root causes, based on the Joint Commission’s taxonomy of five interacting root nodes: impact, type, domain, cause and prevention [44].

## Conclusion

- In this study, more than half of the hospital trauma deaths could have been prevented.
- Airway management, fluid resuscitation and hemorrhage control are the early contributors to death identified in the Indian urban setting.
- Lack of surgical protocols and surgical decision making were identified as systems-related opportunities for improvement.
- Two-thirds of deaths in the mildly injured patients were preventable, and this subgroup is identified for future intervention.

## Abbreviations

ATLS: Advanced trauma life support
DIC: Disseminated intravascular coagulation
DP: Definitely preventable
ISS: Injury severity score
LMIC: Low-middle income countries
NP: Non-preventable
NPI: Non-preventable but care could have been improved
OFI: Opportunities for improvement
PP: Potentially preventable
TITCO-India: towards improving trauma care outcomes in India
WHO: World Health Organization
